# Comparative Diagnostic Accuracy of Ultrasonography and Magnetic Resonance Cholangiopancreatography (MRCP) in the Evaluation of Obstructive Jaundice: A Prospective Study in Western India

**DOI:** 10.7759/cureus.100312

**Published:** 2025-12-29

**Authors:** Parth Katariya, Bhavesh Vaishnani, Hitesh Gamit, Sanjaykumar Vaghela, Krishna Jasani

**Affiliations:** 1 Surgery, P.D.U. Government Medical College, Rajkot, IND; 2 Community and Family Medicine, All India Institute of Medical Sciences, Rajkot, IND

**Keywords:** bile duct dilatation, diagnostic accuracy, mrcp (magnetic resonance cholangiopancreatography), obstructive jaundice, ultrasonography

## Abstract

Background: Obstructive jaundice is a common clinical condition requiring accurate imaging for prompt diagnosis and management. Ultrasonography (USG) is often the first-line modality, whereas magnetic resonance cholangiopancreatography (MRCP) offers non-invasive high-resolution visualization of the biliary and pancreatic ducts. This study aimed to compare the diagnostic accuracy of USG and MRCP in obstructive jaundice and assess the efficacy of MRCP in detecting ancillary biliary and pancreatic abnormalities.

Methods: A hospital-based prospective study was conducted on 30 adult patients (age 18-75 years) with obstructive jaundice at P.D.U. Government Medical College, Rajkot, India, between November 2020 and November 2022. All participants underwent both USG and MRCP. Diagnostic performance was evaluated against the reference standard of intraoperative, histopathological, or endoscopic retrograde cholangiopancreatography findings. Sensitivity, specificity, positive and negative predictive values, accuracy, and Cohen’s kappa coefficient (κ) were calculated. Chi-square test and receiver operating characteristic curve analysis were used to compare modalities.

Results: MRCP demonstrated superior diagnostic performance compared with USG in detecting intrahepatic biliary dilatation (14 (46.7%) vs. 6 (20%), p < 0.05), common bile duct (CBD) dilatation (25 (83.3%) vs. 18 (60%), p < 0.05), and level of obstruction ((26 (86.7%) vs. 19 (63.3%), p = 0.04). For benign biliary pathologies, MRCP achieved higher sensitivity (95.6% vs. 92.3%), specificity (78.9% vs. 50%), and overall accuracy (87.2% vs. 71.1%) compared to USG. In CBD stones, MRCP outperformed USG in sensitivity (96.8% vs. 91.6%), specificity (84.9% vs. 55%), and accuracy (90.8% vs. 73.3%). MRCP demonstrated perfect diagnostic performance in bile duct dilatation, benign strictures, and tumors, with higher concordance to reference standards (κ = 0.92) than USG (κ = 0.56). Overall, MRCP provided more precise detection and characterization of biliary pathology across all parameters.

Conclusion: MRCP significantly outperforms USG in the evaluation of obstructive jaundice, particularly in detecting biliary strictures, ductal dilatation, and tumors. MRCP should be considered the preferred non-invasive diagnostic modality for comprehensive assessment of patients with obstructive jaundice, complementing USG in clinical decision-making.

## Introduction

Obstructive jaundice is a frequent clinical condition resulting from impaired bile flow due to partial or complete blockage of the biliary ducts. The etiology may be intrahepatic, including hepatitis, cirrhosis, or hepatocellular carcinoma, or extrahepatic, encompassing intraductal causes such as choledocholithiasis, benign biliary strictures, and cholangiocarcinoma, as well as extraductal causes such as pancreatic head carcinoma, periampullary tumors, or inflammatory compression secondary to pancreatitis or Mirizzi’s syndrome [1,2]. Among these, choledocholithiasis remains the most prevalent cause of obstructive jaundice globally [3].

Pathophysiologically, obstruction leads to elevated intrabiliary pressure and cholestasis, resulting in regurgitation of conjugated bilirubin into the bloodstream, manifesting clinically as jaundice, dark urine, and clay-colored stools. The biochemical profile typically demonstrates elevated serum bilirubin and alkaline phosphatase, with variable increases in aminotransferases depending on the duration and degree of obstruction [4]. Markedly high bilirubin levels (>20 mg/dL) generally suggest a malignant etiology, whereas moderate elevations are more consistent with benign obstruction such as choledocholithiasis [5].

Accurate identification of the site and cause of obstruction is essential for appropriate therapeutic planning and prognostication. Ultrasonography (USG) remains the first-line investigation due to its wide availability, low cost, and absence of radiation exposure. It has a diagnostic accuracy approaching 90%-95% when bilirubin levels exceed 10 mg/dL [6]. However, USG is operator-dependent and has limited sensitivity in individuals with obesity, patients with excessive bowel gas, or in delineating the distal common bile duct (CBD) and ampullary regions [7].

Magnetic resonance cholangiopancreatography (MRCP), introduced in the early 1990s, has since evolved as a non-invasive, high-resolution imaging technique for evaluating the biliary and pancreatic ductal systems. Using heavily T2-weighted sequences, MRCP provides detailed multiplanar visualization of fluid-filled structures without the need for contrast media [8]. It offers diagnostic accuracy comparable to endoscopic retrograde cholangiopancreatography (ERCP) for many pathologies but without its inherent risks such as pancreatitis, bleeding, or sepsis [9,10]. MRCP also effectively delineates the level of obstruction, assesses biliary anatomy, and characterizes the underlying pathology, making it a valuable alternative to invasive procedures [11]. Despite limitations, including cost, motion artifacts, and contraindications in patients with metallic implants, MRCP has gained prominence as the preferred non-invasive modality for comprehensive biliary evaluation [12].

Given the limitations of USG and the invasive nature of ERCP, there is a growing need to evaluate the comparative diagnostic efficacy of MRCP and USG in detecting biliary obstruction, determining its level, and identifying its etiology. The present study was undertaken to assess the relative accuracy, diagnostic yield, and agreement of MRCP and USG findings in patients with obstructive jaundice, with operative or histopathological findings serving as the reference standard.

Objectives

The objectives of this study are to compare the diagnostic accuracy of USG and MRCP in the evaluation of patients with obstructive jaundice and to evaluate the efficacy of MRCP in diagnosing ancillary findings such as gallstone disease, biliary ductal dilatation, and abnormalities of the pancreatic duct.

## Materials and methods

Study design and setting

This hospital-based, cross-sectional study was conducted in the Department of Surgery, P.D.U. Government Medical College, Rajkot, Gujarat, India, over a two-year period from November 2020 to November 2022.

Sample size

The sample size was determined using formula 4pq/l^2 ^based on feasibility and prior diagnostic accuracy studies comparing USG and MRCP in obstructive jaundice, while ensuring adequate statistical power to detect clinically meaningful differences. Using an expected sensitivity of 85% [12] for MRCP and 65% for USG in detecting the etiology of biliary obstruction, with a two-sided alpha of 0.05 and 80% power, the minimum required sample size for detecting a 20% absolute difference in sensitivity was calculated to be 26 patients. Allowing for up to 15% attrition, a total of 30 patients were enrolled.

Eligibility criteria

Patients were eligible for inclusion if they were between 18 and 75 years of age, had a confirmed diagnosis of obstructive jaundice, and were willing to undergo both USG and MRCP after providing written informed consent. Patients were excluded if they had metallic implants or prosthetic devices that could interfere with MRI, such as cardiac pacemakers, intraocular or cochlear implants, orthopedic joint prostheses, surgical wire sutures, stapes implants, or intrauterine devices. Individuals with claustrophobia or those who declined to participate in the study were also excluded.

A detailed clinical history was obtained from each patient, including symptoms such as right hypochondrial or epigastric pain, jaundice, pruritus, abdominal lump, fever, anorexia, weight loss, clay-colored stools, alcohol intake, prior biliary interventions, and family history of hepatobiliary disease. General and abdominal examinations were performed, noting icterus, pallor, scratch marks, abdominal tenderness, Murphy’s sign, and characteristics of any palpable mass.

Laboratory investigations

Baseline investigations included complete hemogram, liver function tests (serum glutamic pyruvic transaminase, serum glutamic oxaloacetic transaminase, serum bilirubin: direct and indirect, and serum alkaline phosphatase), and urine analysis where indicated.

Imaging protocols

USG

Transabdominal USG was performed using a convex 1-5 MHz probe to evaluate the liver, gallbladder, biliary tree, pancreas, and surrounding abdominal structures. Findings regarding ductal dilatation, level of obstruction, calculus characteristics, and mass lesions were recorded.

MRCP

MRCP was performed on a 1.5 Tesla Achieva (Philips Medical System, Netherlands) using a SENSE body array coil. Patients fasted for at least six hours and removed all metallic objects before imaging. Protocols included 2D and 3D fast spin echo sequences, thin-slab high-resolution (3D HR) acquisitions in axial, coronal, and oblique coronal planes, and thick-slab single-shot MRCP with radial acquisition to encompass the porta hepatis, intrahepatic biliary radicals, and pancreatic duct. Breath-hold and fat-saturation techniques were applied, and source images were reconstructed using maximum intensity projection algorithms with multiplanar and 3D reformatting. Additional T1-weighted, T2-weighted, and contrast-enhanced MRI sequences were performed when indicated.

Reference Standard

The final diagnosis was established based on intraoperative findings, histopathology, or ERCP results, serving as the reference standard.

Data collection tool and statistical analysis

A structured proforma was used to capture demographic details, clinical presentation, laboratory results, and imaging findings from both modalities. All analyses were performed using Microsoft Excel 2013 (Microsoft Corp., Redmond, WA, USA) and jamovi version 2.5.3.0 (jamovi (Version 2.3) [Computer Software]. Retrieved from https://www.jamovi.org). Normality of continuous variables was tested using the Shapiro-Wilk test. Continuous variables were expressed as mean ± standard deviation for normally distributed data and median with interquartile range for skewed distributions; categorical variables were presented as frequencies and percentages. Group comparisons between USG and MRCP for categorical diagnostic parameters were performed using the chi-square test or Fisher’s exact test, based on expected cell frequencies. Diagnostic accuracy was assessed against the reference standard using 2×2 contingency tables to calculate sensitivity, specificity, positive predictive value, negative predictive value, likelihood ratios, and overall accuracy, each with 95% confidence intervals (CIs). Agreement between USG and MRCP was evaluated using Cohen’s kappa coefficient (κ) and interpreted per Landis and Koch benchmarks. Receiver operating characteristic (ROC) curve analysis was performed to compare diagnostic performance, with area under the curve (AUC).

Ethical considerations

Ethical approval was obtained from the Institutional Ethics Committee of P.D.U. Government Medical College, Rajkot. The study was conducted in accordance with the Declaration of Helsinki (2013), and written informed consent was obtained from all participants prior to enrollment.

## Results

A total of 30 patients with obstructive jaundice were included in the present study. The age distribution of patients is shown in Table 1. The majority of patients belonged to the age group of 51-60 years (36.7%), followed by 41-50 years (20%). The youngest patient was 18 years old and the oldest was 75 years old, with a mean age of 52.3 ± 13.8 years. With respect to gender distribution, 16 patients (53.3%) were female and 14 patients (46.7%) were male, giving a male-to-female ratio of 1:1.1. The most common presenting symptom was right upper quadrant pain, which was observed in all patients (100%). Nausea and vomiting were present in 93.3% of patients, while icterus and dark urine/clay-colored stools were each noted in 43.3% of cases. Other less frequent symptoms included weight loss (26.7%), abdominal lump (10%), and fever (6.7%).

**Table 1:** Baseline characteristics of study patients (N = 30) *Multiple responses allowed. ALP, alkaline phosphatase; SD, standard deviation; TLC, total leukocyte count.

Variable	Category	n (%)/mean ± SD
Age group (years)	≤20	1 (3.3)
	21-30	2 (6.7)
	31-40	3 (10.0)
	41-50	6 (20.0)
	51-60	11 (36.7)
	61-70	3 (10.0)
	71-75	4 (13.3)
Gender	Male	14 (46.7)
	Female	16 (53.3)
Symptoms*	Right upper quadrant pain	30 (100.0)
	Nausea/vomiting	28 (93.3)
	Icterus	13 (43.3)
	Dark urine/clay stool	13 (43.3)
	Weight loss	8 (26.7)
	Abdominal lump	3 (10.0)
	Fever	2 (6.7)
Investigations	TLC (/cu mm)	9301.7 ± 3904.5
	Serum ALP (IU/L)	138.17 ± 61.9
	Serum total bilirubin (mg/dL)	2.96 ± 4.16
	Serum conjugated bilirubin (mg/dL)	1.70 ± 2.80

The mean laboratory investigation values of the study cohort are also summarized in Table 1. The average total leukocyte count was 9301.7 ± 3904.5/cu mm. Mean serum alkaline phosphatase was 138.17 ± 61.9 IU/L. The mean serum total bilirubin was 2.96 ± 4.16 mg/dL, with a mean conjugated bilirubin of 1.7 ± 2.8 mg/dL.

Comparative analysis of USG vs. MRC

The diagnostic yield of USG and MRCP for evaluating biliary pathology was compared across multiple parameters. MRCP demonstrated superior diagnostic performance compared with USG in detecting biliary pathology. MRCP identified intrahepatic biliary dilatation in 46.7% of patients compared with 20% detected by USG, a statistically significant difference (χ² = 4.8, df = 1, p < 0.05; risk ratio (RR) = 2.33, 95% CI: 1.07-5.09). Similarly, CBD dilatation was detected in 83.3% of cases by MRCP compared with 60% by USG (χ² = 4.02, df = 1, p < 0.05; RR = 1.39, 95% CI: 1.02-1.89). For combined intrahepatic and CBD dilatation, MRCP showed a markedly higher detection rate (43.3%) than USG (16.7%) (χ² = 5.07, df = 1, p < 0.05), though intermodality agreement was poor (Cohen’s κ = 0.24), indicating frequent underestimation by USG. The level of obstruction was delineated in 86.7% of patients using MRCP compared with 63.3% with USG, with the distribution across porta hepatis, proximal CBD, and distal CBD levels being broadly comparable, but overall detection sensitivity was significantly higher for MRCP (p = 0.04, McNemar test). Regarding the etiology of obstruction, MRCP detected CBD stones in 54.5%, benign strictures in 70%, and tumors in 100% of cases, while USG identified these in 45.5%, 30%, and 100%, respectively. The difference was significant for benign strictures (p < 0.05), with MRCP demonstrating better concordance with operative or histopathological findings (κ = 0.92) than USG (κ = 0.56). Overall, MRCP consistently outperformed USG in accurately detecting and characterizing biliary pathology (Table 2).

**Table 2:** Comparison of USG and MRCP in detection of biliary pathology (N = 30) The table presents the diagnostic yield of USG and MRCP across key biliary parameters. Categorical comparisons were performed using the chi-square test (χ²) or Yates-corrected χ² where appropriate. RR quantifies the relative likelihood of MRCP in detecting a given pathology compared with USG. Cohen’s kappa (κ) indicates the level of agreement between the two modalities for specific etiologies. Sensitivity gain reflects the absolute improvement in MRCP’s ability to identify the level of obstruction. CBD, common bile duct; CI, confidence interval; MRCP, magnetic resonance cholangiopancreatography; RR, risk ratio; USG, ultrasonography.

Diagnostic parameter	USG, n (%)	MRCP, n (%)	Statistical test		
			χ² test	p-value	Effect size/agreement
Intrahepatic biliary dilatation	6 (20.0)	14 (46.7)	χ² = 4.8	0.004	RR = 2.33 (95% CI: 1.07–5.09)
CBD dilatation	18 (60.0)	25 (83.3)	χ² = 4.02	0.01	RR = 1.39 (95% CI: 1.02–1.89)
Both intrahepatic and CBD dilatation	5 (16.7)	13 (43.3)	χ² = 5.07	0.002	Cohen’s κ = 0.24 (poor agreement)
Level of obstruction identified	19 (63.3)	26 (86.7)	Yates χ² = 0.2	0.91	Sensitivity gain +23.4%
– Porta hepatis	2 (10.5)	3 (12.0)			–
– Proximal CBD	5 (26.3)	7 (28.0)			–
– Distal CBD	12 (63.2)	16 (64.0)			–
Etiology of obstruction					
– CBD stone	10 (45.5)	12 (54.5)	χ² = 0.09	0.76	κ = 0.68 (moderate)
– Benign stricture	3 (30.0)	7 (70.0)	χ² = 0.98	0.61	κ = 0.42 (fair)
– Tumor	6 (100.0)	6 (100.0)	χ² = 0.31	0.57	κ = 1.0 (perfect)

In the comparative evaluation of USG and MRCP for benign biliary pathologies, MRCP consistently demonstrated superior diagnostic performance. For benign biliary pathology overall, MRCP achieved higher sensitivity (95.6% vs. 92.3%), specificity (78.9% vs. 50%), and accuracy (87.2% vs. 71.1%) than USG, with a notably higher Youden’s index (74.5% vs. 42.3%). In the diagnosis of CBD stones, MRCP again outperformed USG, showing higher sensitivity (96.8% vs. 91.6%), specificity (84.9% vs. 55%), and overall accuracy (90.8% vs. 73.3%). The Youden’s index was nearly double for MRCP (81.7% vs. 46.6%), highlighting its better balance of sensitivity and specificity. For benign strictures, MRCP showed a sensitivity of 92.6% and perfect specificity (100%), yielding an accuracy of 96.3% and a Youden’s index of 92.6%, compared to 88.5% accuracy and 77.0% index for USG. In bile duct dilatation, MRCP achieved perfect diagnostic performance (100% sensitivity, specificity, accuracy, and Youden’s index), whereas USG also performed well but at slightly lower values (sensitivity 88%, specificity 91%, accuracy 89.5%, Youden’s index 79%). For bile duct tumors, USG demonstrated excellent sensitivity (98%) and specificity (92%) with an accuracy of 95%, while MRCP provided flawless results with 100% across all diagnostic indices, including a Youden’s index of 100%. Overall, MRCP outperformed USG in all categories, particularly in detecting strictures, bile duct dilatation, and tumors, where it achieved perfect or near-perfect diagnostic indices (Table 3).

**Table 3:** Comparative diagnostic performance of USG and MRCP in identifying the level and etiology of obstruction CBD, common bile duct; MRCP, magnetic resonance cholangiopancreatography; NPV, negative predictive value; PPV, positive predictive value; USG, ultrasonography.

Modalities	Sensitivity (%)	Specificity (%)	PPV (%)	NPV (%)	Youden’s index (%)	Accuracy (%)
Detecting benign biliary pathology						
USG	92.3	50.0	91.9	50.0	42.3	71.1
MRCP	95.6	78.9	95.0	78.6	74.5	87.2
Detecting CBD stones						
USG	91.6	55.0	90.0	52.0	46.6	73.3
MRCP	96.8	84.9	96.0	95.0	81.7	90.8
Detecting benign strictures						
USG	85.0	92.0	78.8	78.6	77.0	88.5
MRCP	92.6	100.0	95.0	95.0	92.6	96.3
Detecting bile duct dilatation						
USG	88.0	91.0	82.6	78.8	79.0	89.5
MRCP	100.0	100.0	96.0	100.0	100.0	100.0
Detecting bile duct tumors						
USG	98.0	92.0	85.0	88.8	90.0	95.0
MRCP	100.0	100.0	100.0	100.0	100.0	100.0

**Figure 1:**
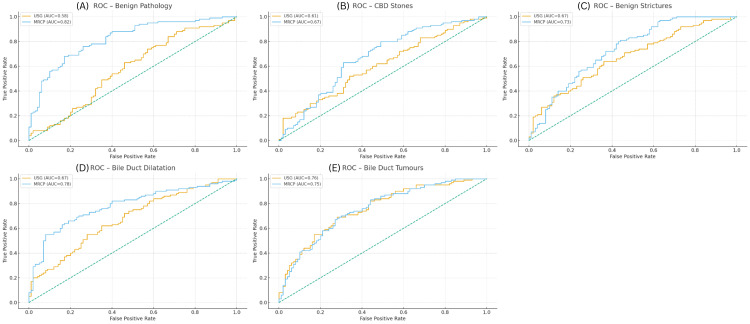
ROC curves of USG and MRCP across major biliary pathologies ROC curves comparing the diagnostic performance of USG and MRCP for six major biliary pathologies: (A) Benign pathology, (B) CBD stones, (C) benign strictures, (D) bile duct dilatation, (E) bile duct tumors, and (F) overall diagnostic accuracy. For each pathology, the true positive rate (sensitivity) is plotted against the false positive rate (1–specificity). The AUC for USG and MRCP is indicated in the legend of each panel. The diagonal dashed line represents the reference line for random classifier performance. MRCP consistently demonstrates higher AUC values than USG across most categories, indicating superior diagnostic accuracy for detecting biliary abnormalities. AUC, area under the curve; CBD, common bile duct; MRCP, magnetic resonance cholangiopancreatography; ROC, receiver operating characteristic; USG, ultrasonography.

The combined multipanel ROC curves (Figures 1A-1E) demonstrate consistently higher AUC values for MRCP compared with USG in four of the five categories, indicating superior overall discriminatory performance. For benign pathology (Figure 1A), MRCP showed markedly better diagnostic accuracy (AUC = 0.82) than USG (AUC = 0.58). In the evaluation of CBD stones (Figure 1B), MRCP again outperformed USG with an AUC of 0.67 vs. 0.61. Similar patterns were observed for benign strictures (Figure 1C), where MRCP achieved an AUC of 0.73 compared with 0.67 for USG. MRCP also demonstrated higher accuracy for bile duct dilatation (Figure 1D), with an AUC of 0.78 vs. 0.67 for USG. In contrast, for bile duct tumors (Figure 1E), the diagnostic performance of both modalities was comparable, with USG showing an AUC of 0.76 and MRCP an AUC of 0.75, suggesting no meaningful difference for this category.

## Discussion

The present study evaluated the diagnostic efficacy of MRCP compared with USG in detecting bile duct abnormalities, using sensitivity, specificity, and predictive values as key performance indicators. Our findings reaffirm the superior diagnostic yield of MRCP in delineating the level and cause of biliary obstruction, particularly in cases of choledocholithiasis, benign strictures, and malignant biliary lesions. In our study, the distal CBD was the most frequent site of obstruction (64%), consistent with previous studies by Arjun Raju et al. [13], Goyani et al. [14], and Al-Obaidi et al. [15], who also reported distal CBD as the predominant level of obstruction. However, Upadhyaya et al. [16] observed the intrapancreatic portion of the CBD as the most common site (38%), possibly due to differing etiological spectra and imaging criteria in their study population.

The most common cause of obstruction in our cohort was CBD stones, aligning with the findings of Kaur et al. [17]. While studies by Arjun Raju et al. [13], Upadhyaya et al. [16], and Madhok et al. [18] reported pancreaticobiliary tumors as the leading cause, and Rathore et al. [19] observed choledocholithiasis as most prevalent, these variations likely reflect geographic and referral biases, as well as differences in patient selection criteria.

Regarding diagnostic performance, MRCP demonstrated high sensitivity (96.8%) and specificity (84.9%) for CBD stone detection, in concordance with previous studies by Soto et al. [20] (sensitivity 94%, specificity 100%) and Pavone et al. [21] (sensitivity 88.9%, specificity 100%). Similar accuracy rates were reported by Regan et al. [22] and Hazem et al. [23], further validating MRCP’s reliability in identifying biliary calculi. MRCP effectively visualized calculus regions as signal voids against the high T2-weighted biliary background, enhancing its diagnostic clarity. For benign biliary strictures, MRCP achieved sensitivity and specificity of 92.6% and 100%, respectively, which is comparable to the results reported by Al-Obaidi et al. [15] (sensitivity 100%, specificity 98.5%), Munir et al. [24] (sensitivity 83.3%, specificity 97.6%), and Bhatt et al. [25] (sensitivity 100%). The high-performance metrics observed reinforce MRCP’s capacity to identify and characterize benign ductal narrowing, particularly in cases where USG findings are inconclusive due to bowel gas or limited acoustic access. In evaluating benign biliary pathologies, MRCP exhibited a sensitivity of 95.6% and specificity of 78.9%, findings consistent with Kurian et al. [26] (sensitivity 94.4%, specificity 50%), Kaur et al. [17], and Singh et al. [27] (sensitivity 100%). MRCP’s ability to accurately delineate both intra- and extrahepatic biliary dilatation makes it a valuable non-invasive alternative to diagnostic ERCP. For tumor detection, MRCP showed excellent diagnostic performance with both sensitivity and specificity of 100%, corroborating the observations of Kaur et al. [17] and Kushwah et al. [28]. These results affirm MRCP’s role as a precise imaging modality for identifying malignant obstructions, while simultaneously offering superior visualization of the biliary anatomy and surrounding structures compared with USG.

MRCP has several established advantages: it is non-invasive, radiation-free, and less operator-dependent than USG, while providing better visualization of ducts proximal to the obstruction. Moreover, when combined with conventional T1- and T2-weighted sequences, MRCP can detect extraductal disease, offering a comprehensive overview of hepatobiliary pathology. Despite its benefits, MRCP’s limitations include contraindications in patients with metallic implants, pacemakers, or claustrophobia, and its inability to provide therapeutic intervention. Nonetheless, its high diagnostic accuracy, safety, and reproducibility make it a compelling first-line imaging choice for evaluating biliary and pancreatic ductal disorders, potentially reducing the need for invasive ERCP procedures.

This study has several limitations that should be acknowledged. First, the sample size was relatively small (N = 30), which may limit the statistical power and generalizability of the findings to broader populations with obstructive jaundice. Second, as a single-center study conducted in a tertiary hospital, referral bias may have influenced the spectrum of cases, particularly the proportion of malignant vs. benign etiologies. Third, although MRCP and USG findings were compared with a reference standard, not all patients underwent the same verification procedure (operative confirmation, ERCP, or histopathology), which may introduce verification bias. Fourth, operator dependency of USG, particularly variability in expertise and technique, may have affected diagnostic accuracy, but interobserver reliability was not assessed. Fifth, MRCP acquisition parameters were standardized; however, variations in patient cooperation, breath-holding ability, and motion artefacts could have influenced image quality and diagnostic interpretation. Finally, because the study focused primarily on diagnostic performance, other clinically relevant parameters such as cost-effectiveness, accessibility, and time to diagnosis were not evaluated, limiting the application of findings in resource-constrained settings.

## Conclusions

Early and accurate diagnosis of biliary obstruction is critical for guiding management and improving patient outcomes. While USG remains a widely available screening tool, its operator dependency and limited field of view may result in underdiagnosis, particularly in complex or distal ductal pathologies. MRCP, with its superior spatial resolution and multiplanar imaging capabilities, bridges this diagnostic gap by offering comprehensive visualization of both the level and etiology of obstruction. Therefore, the present study underscores the need for adopting MRCP as a primary diagnostic tool in patients with suspected biliary obstruction, reserving invasive procedures such as ERCP for therapeutic purposes.
